# Altered mental status in a 5‐month‐old boy

**DOI:** 10.1002/ccr3.2932

**Published:** 2020-05-24

**Authors:** Shafee Salloum

**Affiliations:** ^1^ Department of Pediatric Hospital Medicine Dayton Children's Hospital Dayton OH USA

**Keywords:** enema, hematochezia, intussusception, lethargy

## Abstract

Intussusception in infants can present early as lethargy or altered mental status alone without apparent gastrointestinal manifestations. Awareness of this condition can result in early diagnosis and prevention of further complications.

## Graphical abstract

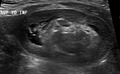

## 1 PRESENTATION

A 5‐month‐old previously healthy boy presented to outside emergency department (ED) with sudden onset of lethargy described as “being drowsy and limp with shallow breathing” lasting for one minute followed by intermittent crying and sleepiness. He was in his normal state of health earlier that day, feeding well, and had no fever, vomiting, or diarrhea. There were no known sick contacts. He was born full‐term at home with no prenatal or postnatal care and was unimmunized due to parental beliefs. His growth and developmental milestones were age appropriate. He had two episodes of body stiffness and unresponsiveness at the outside ED, thought to be seizures in nature, each one lasted for about 30 seconds. Serum glucose was 102 mg/dL (normal). He received intravenous (IV) levetiracetam and was transferred to our facility. He developed one episode of nonbilious emesis en route.

**Figure 1:**
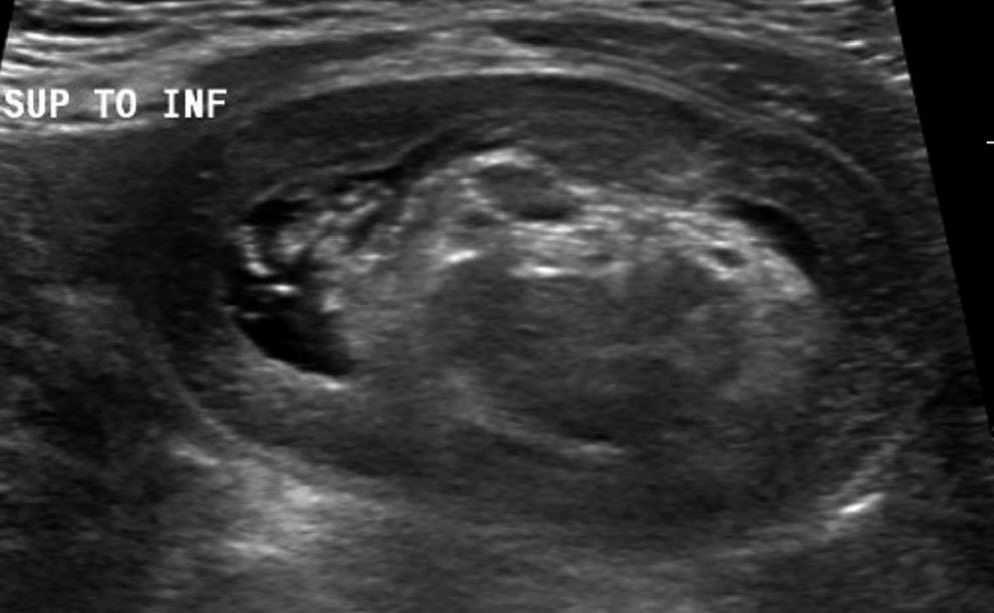
Abdominal ultrasonography shows target sign due to large ileocolic intussusception

On physical examination in our ED, temperature 37.1°C, heart rate 117 beats/minute, blood pressure 98/57 mm Hg, respiratory rate 36 breaths/minute, and oxygen saturation 99% on room air were observed. He appeared pale and somnolent. His pupils were 3 mm and reactive to light, and his anterior fontanel was soft and flat. His abdomen was soft, nondistended with no palpable masses or organomegaly. Capillary refill time was <2 seconds. The rest of his examination was unremarkable with no signs of trauma. An extensive workup was performed to find out the reason for his lethargy and seizure‐like activity. Laboratory evaluation revealed normal electrolytes, glucose, and renal function. White blood cell count was 14 800/μL; otherwise, complete blood count was unremarkable. Head computed tomography, cerebrospinal fluid analysis, electrocardiogram, and urine toxicology were all unremarkable. IV antibiotics were administered, and electroencephalogram (EEG) was performed prior to admission to the hospital. Five hours after admission, he passed bloody stool for which an abdominal sonography was obtained and it revealed large ileocolic intussusception (Figure 1).

## 2 DIFFERENTIAL DIAGNOSIS

The differential diagnosis of lethargy in infants includes infection causes like meningitis, encephalitis, and sepsis, accidental and nonaccidental trauma, metabolic derangements, hypoglycemia, intoxication, and as we saw in this case, intussusception.

## 3 TREATMENT

Pneumatic enema reduction was performed which revealed intussusception extending to the sigmoid colon. The patient had three subsequent intussusception recurrences, and ultimately he required diagnostic laparoscopy with manual reduction of his intussusception. No pathologic lead point could be identified. He tolerated the surgery well and was discharged home on the next day. He was healthy at 1‐month follow‐up visit.

## 4 DISCUSSION

Intussusception is the most common cause of bowel obstruction in infancy and early childhood, and the majority of cases occur before 2 years of age.^1^, ^2^ It results from telescoping of one bowel segment into the distal segment, most commonly the ileum into the cecum (ileocecal) bringing the mesentery with it, causing obstruction of the venous return and bowel engorgement and ischemia which in turn results in hematochezia. This hematochezia mixed with mucous gives the classical, but usually late, finding of currant jelly stools. If left untreated, this can lead to bowel perforation, peritonitis, and ultimately death.^1^, ^2^

Intussusception is usually idiopathic in up to 90% of cases with no identified pathologic lead point. Inflamed Peyer's patches in the terminal ileum secondary to a viral infection are believed to be the lead point in idiopathic cases. Pathologic lead points are more common in children older than 3 years of age, and these include Meckel diverticulum, intestinal polyps, duplication cysts, lymphoma, and hemangioma.^1^, ^2^, ^3^, ^4^ Iatrogenic small bowel intussusceptions have been reported in children who were fed with nasojejunal or gastrojejunal feeding tubes.^5^, ^6^

The typical presentation of intussusception is a sudden onset colicky abdominal pain in a previously well and healthy child. Initially, patient may be asymptomatic between these episodes, but as the intussusception persists, this can lead to progressive weakness and lethargy. Emesis occurs in most cases, initially nonbilious, but later becomes bile stained due to bowel obstruction. Stool is usually guaiac positive, and gross blood becomes evident later. The classical triad of colicky abdominal pain, palpable sausage‐shaped abdominal mass, and hematochezia is present in only one third of cases.^1^, ^2^

Altered level of consciousness or lethargy might be the predominant and the only presenting feature of intussusception without apparent gastrointestinal manifestations, especially in infants.^7^ One would expect depressed level of consciousness in late presentation with prolonged symptoms resulting in septic shock and bowel perforation. However, lethargy early in the course of the illness when the patient is still hemodynamically stable cannot be explained by these factors. The exact pathophysiology behind this early lethargy in intussusception is unknown. One proposed mechanism suggests endogenous opioids release in response to severe abdominal pain supported by positive response to naloxone infusion.^8^ The two episodes of body stiffness and unresponsiveness described at the outside ED in this patient could be a normal infant response to severe pain similar to the classic description of “drew up his knees” in older children with intussusception.

Abdominal sonography is the gold standard for diagnosis of intussusception, revealing the classic target or donut appearance in the cross‐sectional view and pseudo‐kidney or tubular mass appearance in the longitudinal view. Ultrasound has almost 100% sensitivity and specificity. Abdominal x‐ray may show a mass or density in the area of intussusception and gas paucity in the right lower quadrant, but is not diagnostic. It is usually helpful if perforation is suspected as it shows pneumoperitoneum.^1^, ^2^, ^3^ Computed tomography CT of abdomen is usually not indicated. Nonsurgical reduction can be achieved with air, saline, or water‐soluble contrast enema under fluoroscopy or sonography guidance. Air enema reduction has gained popularity in recent years as it avoids peritoneal irritation in cases of perforation, is cleaner, and less expensive. Enema reduction is contra‐indicated in late presentation with signs and symptoms of perforation and peritonitis. Pediatric surgery should be consulted even in stable patients undergoing enema reduction in case of complications and the need for emergent laparotomy.^2^, ^3^

## CONFLICT OF INTEREST

None declared.
